# Effects of dietary supplementation of probiotic, *Clostridium butyricum,* on growth performance, immune response, intestinal barrier function, and digestive enzyme activity in broiler chickens challenged with *Escherichia coli* K88

**DOI:** 10.1186/s40104-016-0061-4

**Published:** 2016-01-26

**Authors:** Ling Zhang, Lingling Zhang, Xiu’an Zhan, Xinfu Zeng, Lin Zhou, Guangtian Cao, An’guo Chen, Caimei Yang

**Affiliations:** College of Animal Sciences, Zhejiang University, Hangzhou, 310058 China; College of Animal Science and Technology, Zhejiang A & F University, 88 North Huancheng Road, Lin’an, Zhejiang 311300 China; Zhejiang Huijia Biological Technology Ltd., Anji, 313307 China

**Keywords:** Broiler chickens, *Clostridium butyricum*, Digestive enzyme activity, *Escherichia coli* K88, Growth performance, Immune response, Intestinal barrier

## Abstract

**Background:**

Colibacillosis caused by enterotoxigenic *Escherichia coli* (*E. coli*) results in economic losses in the poultry industry. Antibiotics are usually used to control colibacillosis, however, *E. coli* has varying degrees of resistance to different antibiotics. Therefore the use of probiotics is becoming accepted as an alternative to antibiotics. In this study, we evaluated the effects of *Clostridium butyricum* (*C. butyricum*) on growth performance, immune response, intestinal barrier function, and digestive enzyme activity in broiler chickens challenged with *Escherichia coli* (*E. coli*) K88.

**Methods:**

The chickens were randomly divided into four treatment groups for 28 days. Negative control treatment (NC) consisted of birds fed a basal diet without *E. coli* K88 challenge and positive control treatment (PC) consisted of birds fed a basal diet and challenged with *E. coli* K88. *C. butyricum* probiotic treatment (CB) consisted of birds fed a diet containing 2 × 10^7^ cfu *C. butyricum*/kg of diet and challenged with *E. coli* K88. Colistin sulfate antibiotic treatment (CS) consisted of birds fed a diet containing 20 mg colistin sulfate/kg of diet and challenged with *E. coli* K88.

**Results:**

The body weight (BW) and average day gain (ADG) in the broilers of CB group were higher (*P* < 0.05) than the broilers in the PC group overall except the ADG in the 14-21 d post-challenge. The birds in CB treatment had higher (*P* < 0.05) concentration of tumor necrosis factor-α (TNF-α) at 3 and 7 d post-challenge, and higher (*P* < 0.05) concentration of interleukin-4 (IL-4) at 14 d post-challenge than those in the PC treatment group. The concentration of serum endotoxin in CB birds was lower (*P* < 0.05) at 21 d post-challenge, and the concentrations of serum diamine oxidase in CB birds were lower *(P < 0.05)* at 14 and 21 d post-challenge than in PC birds. Birds in CB treatment group had higher (*P* < 0.05) jejunum villi height than those in PC, NC, or CS treatment at 7, 14, and 21 d post-challenge. In comparison to PC birds, the CB birds had lower (*P* < 0.05) jejunum crypt depth during the whole experiment. The birds in CB or CS treatment group had higher (*P* < 0.05) activities of amylase and protease at 3, 7, and 14 d post-challenge, and higher (*P* < 0.05) activity of lipase at 3, 7 d post-challenge than PC birds.

**Conclusions:**

In all, these results indicate that dietary supplementation with *C. butyricum* promotes immune response, improves intestinal barrier function, and digestive enzyme activities in broiler chickens challenged with *E. coli* K88. There is no significant difference between the *C. butyricum* probiotic treatment and the colistin sulfate antibiotic treatment. Therefore, the *C. butyricum* probiotic may be an alternative to antibiotic for broiler chickens.

## Background

Colibacillosis caused by enterotoxigenic *Escherichia coli* (*E.coli*) is a serious infection that results in huge economic losses in the poultry industry worldwide [1–4]. Although antibiotics are usually used to control colibacillosis, various reports have demonstrated that pathogenic *E. coli* has varying degrees of resistance to different antibiotics [5, 6]. Additionally, resistance genes extended-spectrum beta-lactamases (ESBL) and/or plasmid-mediated Amp-C beta-lactamases (Amp-C) in commercial *E. coli* may pose a human health hazard. [7] Therefore, there is an urgent need to identify sustainable alternatives to antibiotics for animal production.

The use of probiotics in the poultry industry is quickly becoming accepted as a potential alternative to antibiotics for use as growth-promoters, and in some cases, for control of specific enteric pathogens [8–15].

*Clostridium butyricum* (*C. butyricum*) is a butyric-acid producing Gram-positive anaerobe found in soil and intestines of healthy animals and humans. *C. butyricum* increases the concentrations of n-butyric acid in caecaldigesta of birds [16], and butyric acid is of particular importance because of its nutritional properties for epithelial cells and pathogen inhibitory effects in the gut [17]. *C. butyricum* also survives at low pH and high temperature, which renders it a good feed additive [18]. Previous studies demonstrated that *C. butyricum* promoted growth performance [16, 19–21], balanced intestinal microflora [16, 17, 19, 20], improved intestinal morphology [16, 19], stimulated the immune system [19, 20], improved meat quality and fatty acid profiles [21–23], and influenced the digestive tract [23] in broiler chickens. In addition, *C. butyricum* prevented *E.coli*-induced intestinal disorders through inhibiting *E.coli* viability and mediating *E.coli*-induced apoptosis [24]. However, there are few published reports on the effects of *C. butyricum* on *E.coli*-challenged animals. The present study was conducted to investigate the effects of *C. butyricum* on the immune response, intestinal barrier function, and digestive enzyme activities in broiler chickens challenged with *E. coli* K88.

## Methods

### Ethics statement

All procedures were approved by the Institutional Animal Care and Use Committee of Zhejiang University.

### Birds, diets, and experimental design

Three hundred and sixty 1-d-old male Cobb broiler chickens purchased from a commercial hatch (Charoen Pokphand Group, Haining, China) were randomly assigned to four treatment groups. Negative control treatment (NC) consisted of birds fed a basal diet without challenging with *E. coli* K88. Positive control treatment (PC) consisted of birds fed a basal diet and orally challenged with 0.5 mL *E. coli* K88 (2 × 10^8^ cfu/mL) on d 7. The *C. butyricum* treatment (CB) probiotic group consisted of birds fed a diet containing 2 × 10^7^ cfu *C. butyricum*/kg of diet and orally challenged with 0.5 mL *E. coli* K88 (2 × 10^8^ cfu/mL) on d 7. Colistin sulfate treatment (CS) antibiotic group consisted of birds fed a diet containing 20 mg colistin sulfate/kg of diet and orally challenged with 0.5 mL *E. coli* K88 (2 × 10^8^ cfu/mL) on d 7. Each treatment consisted of 6 replicate pens with 15 birds per pen. Birds in NC treatment were housed in one room, while the birds in other three *E. coli*-challenged treatment groups were housed in another room to prevent cross-contamination. The two rooms were of the same configuration and the previous growth studies revealed no significant contamination room effects.

Chickens were placed in the wire cages and all birds were offered the same antibiotic-free basal diets and provided ad libitum access to water and diet. The nutrient levels of the diets met the NRC (1994) broiler recommendations (Table 1). The temperature was adjusted to 32 °C in the first week and gradually lowered to 25 °C.

**Table 1:** The composition and nutrients of basal diet^a^

Ingredient	Content, %	Chemical composition	Content
Corn	55.23	CP, %	20.90
Soybean meal	30.67	ME, Mcal/kg	3.00
Wheat shorts	4.00	Calcium, %	1.00
Fish meal^b^	3.00	Total P, %	0.65
Soybean oil^c^	2.90	Available P, %	0.45
DL-Methionine	0.27	Methionine + cysteine, %	0.90
NaCl	0.27	Lysine, %	1.05
Limestone	1.33		
Calcium phosphate	1.33		
Vitamin-mineral premix^d^	1.00		

^a^Nutrient level of the diets was based on NRC (1994)

^b^Crude protein content is 62.5% and metabolizable energy is 2.79 Mcal/kg

^c^Metabolizable energy is 8.8 Mcal/kg

^d^Supplied per kilogram of diet: vitamin A(retinyl acetate), 1,500 IU; cholecalciferol, 200 IU; vitamin E(DL-α-tocopheryl acetate), 10 IU; riboflavin, 3.5 mg; pantothenic acid, 10 mg; niacin, 30 mg; cobalamin, 10μg; choline chloride, 1,000 mg; biotin, 0.15 mg; folic acid, 0.5 mg; thiamine 1.5 mg; pyridoxine 3.0 mg; Fe, 80 mg; Zn, 40 mg; Mn, 60 mg; I, 0.18 mg; Cu, 8 mg; Se, 0.15 mg

The *C. butyricum* strain (HJCB998) was obtained from Zhejiang Huijia Biological Technology Ltd., Anji, China. The probiotic strain was grown anaerobically in a liquid fermentation tank at 37 °C for 48 h, and then the cells were harvested by centrifugation and dried by spray-drying technology. Colistin sulfate was obtained from Zhejiang Qianjiang Biochemical Ltd., Haining, China.

### Oral challenge

The *E. coli* K88 strain was obtained from College of Animal Sciences, Zhejiang University (Hangzhou, China) and grown at 37 °C. The birds in different treatments were fed the corresponding diet for the first 6 d. On d 7, birds in PC, CB, and CS treatments were orally fed with 0.5 mL (2 × 10^8^ cfu/mL) *E. coli* K88 inoculants by using a polyethylene tube attached to a syringe. The birds in NC treatment were administered the same amount of sodium chloride solution as control.

### Sample collection

Birds were weighed individually at 3, 7, 14, and 21 d post-challenge to evaluate BW and ADG. Feed consumption and feed-to-gain ratio could not be determined because of an indeterminate amount of feed wastage.

Six birds per treatment (1 bird per pen) were randomly selected for sample collection at 3, 7, 14, and 21 d post-challenge. Blood samples were taken from the wing vein and centrifuged (3,000 × g, 10 min) at 4 °C, and then the serum was harvested and stored at -20 °C until analysis. The birds were then killed by CO_2_ inhalation and jejunum samples were collected. Two 1 cm segments of jejunum were collected immediately after slaughter. The segment was located in a distal segment about 5 cm proximal to the duodenum. The surplus jejunum section was gently flushed with phosphate-buffered saline (PBS) and the mucosa was scraped from the jejunum with a sterile blade and stored in a 1.5 mL sterile microcentrifuge tube at -20 °C.

### Mucosal cytokines

Jejunal mucosa (0.5 g per sample) was weighed out, diluted into 4.5 mL of 0.9 % salt solution, and centrifuged at 6,000× g for 15 min. The homogenate was kept on sterile ice and the supernatant was harvested into 1.5 mL sterile microcentrifuge tubes. The concentrations of interleukin-4 (IL-4) and tumor necrosis factor-α (TNF-α) were respectively measured using IL-4 (1042-09) and TNF-α (1041-09) ELISA kits (GBD Ltd, USA) specific for chicken.

### Serum endotoxin and diamine oxidase

The concentrations of serum endotoxin were measured using a limulus amoebocyte lysate (LAL)-based kit (LAL QCL-1000 kit, Lonza, Walkersville, MD). The samples were heated for 10 min at 70 °C. Internal control for recovery calculation was included in the assessment. Standards and samples were incubated for 10 min at 37 °C with LAL and then for another 6 min with colorimetric substrate. The reaction was stopped with 25 % acetic acid and then the absorbance was read at 405 nm. Diamine oxidase (DAO) activity (1 ml) was examined by a spectrophotometric assay. The DAO standard (batch number D7876-250) was purchased from Sigma.

### Jejunalmorphology analysis

Jejunal segments were flushed with a 0.9 % salt solution, and then fixed with 10 % formaldehyde-phosphate buffer for 48 hours. The formalin-fixed, paraffin-embedded tissues were embedded into Leica EG1160, fixed upon Rotary Microtome (Leica RM2153) and then cut to a thickness of 6 μm. The tissue segments were dehydrated with Leica HI1220. Slides were stained with hematoxylin and eosin (H&E; Leica Autostain BRXL) and covered by cover slides. Images were analyzed using software Qwin. Then the 10 longest jejunal villi and lowest jejunal crypts were measured with Olympus AX70 microscope (Olympus Corporation, Tokyo, Japan) and the mean value was calculated. The villus height was measured from the villus-crypt junction to the tip of villus, whereas crypt depth was measured from the root of villus to the lamina propria.

### Digestive enzyme activities

Amylase, lipase, and protease were analyzed using the corresponding kit provided by Jiancheng Bioengineering Institute (Nanjing, China). In brief, the jejunal mucosa was transferred into sterilized tubes containing 10 mL PBS (7.4 pH), then ultrasonic treatment was applied for 4 min to dissociate the tissues. The later procedure was accomplished by centrifugation (5,000 × g for 25 min). Then the supernatant was used to determine the enzymatic activities following the manufacturer’s instructions.

### Statistical analysis

One-way ANOVA was performed using SPSS 16.0 Software. Mean values of treatment groups were compared using Duncan’s multiple range test with *P* < 0.05 considered statistically significant.

## Results

Birds in the PC (positive control) treatment group had less (*P* < 0.05) BW than the NC (negative control), CB (*C. butyricum*), and CS (colistin sulfate) birds from 3 to 21d post-challenge (Table 2). There were no significant differences among the BW of NC, CB, and CS groups. The birds of CB group had higher ADG than those fed the PC diet during 3-7, 7-14, and 3-21 d post-challenge. No significant differences in BW and ADG were observed among the birds of NC, CB, and CS groups except that the ADG of CB and CS broilers was higher than the broilers of the NC groups in 3-7 d post-challenge.

**Table 2:** Effects of *Clostridium butyricum* on growth performance in broilers^1^

Items	Age of (post-ch)^2^	Experimental treats				Statistics	
		NC	PC	CB	CS	SEM	*P*-value
BW, g	3d	351.71^a^	319.83^b^	342.23^a^	339.95^a^	3.588	<0.01
	7d	401.16^a^	354.00^b^	402.71^a^	398.81^a^	4.479	<0.01
	14d	747.33^ab^	649.00^c^	774.83^a^	738.16^b^	11.161	<0.01
	21d	1283.5^a^	1064.8^b^	1265.8^a^	1275.2^a^	26.491	<0.01
ADG, g	3-7d	12.36^b^	8.54^c^	15.12^a^	14.71^a^	0.651	<0.01
	7-14d	49.45^a^	42.14^b^	53.16^a^	48.47^a^	1.117	<0.01
	14-21d	76.60	59.40	70.13	76.72	2.831	0.089
	3-21d	51.76^a^	41.38^b^	51.30^a^	51.95^a^	1.365	<0.01

^a-c^Means in the same row with different superscript letters differ significantly (*P* < 0.05)

^1^Each mean represents 6 birds. NC = birds fed a basal diet without challenged with *E. coli* K88; PC = birds fed a basal diet and challenged with *E. coli* K88. CB = birds fed a basal diet including 2 × 10^7^ CFU *C. butyricum*/kg of diet and challenged with *E. coli* K88. CS = birds fed a basal diet including 20 mg colistin sulfate/kg of diet and challenged with *E. coli* K88

^2^The days after challenging

Birds in CB treatment had higher (*P* < 0.05) concentration of jejunal mucosa TNF-α than those in NC or PC treatment at 3 d post-challenge, and higher (*P* < 0.05) concentration of TNF-α than PC birds at 7 d post-challenge (Table 3). There was no significant difference in the concentration of TNF-α between CB and CS birds during the whole experiment. In comparison to PC birds, CB birds had greater (*P* < 0.05) concentration of jejunal mucosa IL-4 on 14 d post-challenge. No significant differences were observed in the concentration of IL-4 among CB, NC, and CS treatments during the whole experiment.

**Table 3:** Effects of *Clostridium butyricum* on jejunal mucosa cytokines in broilers^1^

Items	Age of (post-ch)^2^	Experimental treats				Statistics	
		NC	PC	CB	CS	SEM	*P*-value
TNF-α, ng/L	3d	53.80^b^	48.88^b^	76.66^a^	65.09^ab^	7.19	0.030
	7d	65.29^ab^	53.88^b^	69.32^a^	61.51^ab^	4.72	0.040
	14d	63.39	50.22	62.92	61.23	6.88	0.220
	21d	50.56	35.28	54.46	56.92	6.92	0.110
IL-4, ng/L	3d	52.52	50.9	68.7	67.51	5.78	0.140
	7d	68.23^a^	52.16^b^	62.91^ab^	57.03^ab^	3.55	0.030
	14d	70.65^a^	50.46^b^	69.79^a^	59.78^ab^	4.26	0.010
	21d	52.64	42.59	56.38	57.09	6.41	0.350

^a-c^Means in the same row with different superscript letters differ significantly (*P* < 0.05)

^1^Each mean represents 6 birds. NC = birds fed a basal diet without challenged with *E. coli* K88; PC = birds fed a basal diet and challenged with *E. coli* K88. CB = birds fed a basal diet including 2 × 10^7^ CFU *C. butyricum*/kg of diet and challenged with *E. coli* K88. CS = birds fed a basal diet including 20 mg colistin sulfate/kg of diet and challenged with *E. coli* K88

^2^The days after challenging

The *E. coli* challenge significantly increased the concentration of serum endotoxin during the whole experiment (Table 4). Birds in CB treatment had lower (*P* < 0.05) serum endotoxin at 21d post-challenge compared with PC birds. There were no significant differences in the concentrations of serum endotoxin between CB and CS treatment during the whole experiment. The *E. coli* challenge significantly increased the concentration of serum DAO during the entire experimental period. Birds in CB treatment had lower (*P* < 0.05) concentration of serum DAO than those in PC treatment at 14 and 21 d post-challenge. No significant differences were found in the concentration of serum DAO between CB and CS treatment group during the course of the experiment.

**Table 4:** Effects of *Clostridium butyricum* on the concentrations of serum LPS and DAO in broilers^1^

Items	Age of (post-ch)^2^	Experimental treats				Statistics	
		NC	PC	CB	CS	SEM	*P*-value
Endotoxin, EU/mL	3d	0.460^b^	0.738^a^	0.704^a^	0.734^a^	0.025	<0.01
	7d	0.455^b^	0.640^a^	0.586^a^	0.578^a^	0.027	<0.01
	14d	0.327^b^	0.413^a^	0.335^ab^	0.347^ab^	0.024	0.070
	21d	0.252^c^	0.380^a^	0.304^bc^	0.332^ab^	0.023	<0.01
DAO, U/mL	3d	2.559^b^	8.823^a^	7.493^a^	8.056^a^	0.498	<0.01
	7d	1.570^b^	8.649^a^	7.121^a^	7.496^a^	0.617	<0.01
	14d	1.250^c^	6.254^a^	4.194^b^	4.201^b^	0.527	<0.01
	21d	0.819^c^	3.952^a^	2.419^b^	3.060^ab^	0.424	<0.01

^a-c^Means in the same row with different superscript letters differ significantly (*P* < 0.05)

^1^Each mean represents 6 birds. NC = birds fed a basal diet without challenged with *E. coli* K88; PC = birds fed a basal diet and challenged with *E. coli* K88. CB = birds fed a basal diet including 2 × 10^7^ CFU *C. butyricum*/kg of diet and challenged with *E. coli* K88. CS = birds fed a basal diet including 20 mg colistin sulfate/kg of diet and challenged with *E. coli* K88

^2^The days after challenging

Birds fed CB had higher (*P* < 0.05) jejunum villi height than PC, NC, or CS birds at 7, 14, and 21 d post-challenge (Table 5). In comparison to the broilers in PC treatment, broilers in CB treatment had lower (*P* < 0.05) jejunum crypt depth throughout the experiment. Birds fed CS had lower (*P* < 0.05) jejunum crypt depth compared to PC birds at 7, 14, and 21 d post-challenge.

**Table 5:** Effects of *Clostridium butyricum* on Jejunum morphometry in broilers^1^

Items	Age of (post-ch)^2^	Experimental treats				Statistics	
		NC	PC	CB	CS	SEM	*P*-value
Villi height, μm	3d	264.35	259.81	275.71	253.01	5.69	0.220
	7d	267.65^bc^	287.54^b^	346.75^a^	254.41^c^	7.91	<0.01
	14d	397.49^b^	355.07^c^	448.51^a^	410.9^b^	8.24	<0.01
	21d	429.41^b^	433.6^b^	531.09^a^	407.26^b^	9.37	<0.01
Crypt depth, μm	3d	50.22^a^	47.01^ab^	33.68^c^	42.9^b^	2.08	<0.01
	7d	50.06^b^	60.3^a^	39.44^c^	38.2^c^	2.17	<0.01
	14d	66.07^b^	73.96^a^	56.51^c^	59.95^c^	1.87	<0.01
	21d	82.5^b^	115.46^a^	84.04^b^	76.03^b^	3.29	<0.01

^a-c^Means in the same row with different superscript letters differ significantly (*P* < 0.05)

^1^Each mean represents 6 birds. NC = birds fed a basal diet without challenged with *E. coli* K88; PC = birds fed a basal diet and challenged with *E. coli* K88. CB = birds fed a basal diet including 2 × 10^7^ CFU *C. butyricum*/kg of diet and challenged with *E. coli* K88. CS = birds fed a basal diet including 20 mg colistin sulfate/kg of diet and challenged with *E. coli* K88

^2^The days after challenging

The *E. coli* challenge significantly decreased the activity of jejunal mucosa amylase; however, broilers fed with CB or CS had increased (*P* < 0.05) amylase activity compared with broilers in the PC treatment group from 3 to 14 d post-challenge (Table 6). No significant differences were found in the activities of amylase between CB and CS treatments during the whole experiment. Compared with PC birds, the birds in CB or CS treatments had higher (*P* < 0.05) activities of protease from 3 to 14 d post-challenge. There were no significant differences in the activity of protease among the four treatment groups at 21 d post-challenge. Moreover, there were no significant differences between CB and NC treatments in the activity of lipase at 3 and 7 d post-challenge, but those two treatment groups had higher (*P* < 0.05) activity of lipase than PC treatment; and no significant differences in the activity of lipase among the four treatments at 14 and 21 d post-challenge.

**Table 6:** Effects of *Clostridium butyricum* on digestive enzyme activities in broilers^1^

Items	Age of (day post-ch)^2^	Experimental treats				Statistics	
		NC	PC	CB	CS	SEM	*P*-value
Amylase, U/mgprot	3d	0.94^a^	0.33^c^	0.70^b^	0.74^ab^	0.058	<0.01
	7d	0.96^a^	0.45^b^	0.88^a^	0.83^a^	0.054	<0.01
	14d	0.80^a^	0.60^b^	0.87^a^	0.78^a^	0.033	0.018
	21d	0.91^a^	0.75^b^	0.79^ab^	0.85^ab^	0.027	0.112
Protease, U/mgprot	3d	106.86^a^	54.66^c^	76.92^b^	80.10^b^	5.080	<0.01
	7d	103.76^a^	65.29^b^	93.32^a^	89.39^a^	4.213	<0.01
	14d	98.17^a^	67.04^b^	93.60^a^	91.46^a^	3.975	0.014
	21d	130.76	109.06	133.03	125.01	4.648	0.264
Lipase, U/mgprot	3d	190.87^ab^	105.14^c^	154.02^b^	205.53^a^	10.833	<0.01
	7d	193.84^a^	105.66^b^	186.09^a^	155.90^ab^	10.800	<0.01
	14d	183.76	167.93	205.40	195.76	7.046	0.278
	21d	194.07	177.85	197.87	203.41	5.697	0.448

^a-c^Means in the same row with different superscript letters differ significantly (*P* < 0.05)

^1^Each mean represents 6 birds. NC = birds fed a basal diet without challenged with *E. coli* K88; PC = birds fed a basal diet and challenged with *E. coli* K88. CB = birds fed a basal diet including 2 × 10^7^ CFU *C. butyricum*/kg of diet and challenged with *E. coli* K88. CS = birds fed a basal diet including 20 mg colistin sulfate/kg of diet and challenged with *E. coli* K88

^2^The days after challenging

## Discussion

Many reports have showed that probiotics can promote growth performance and improve nutrient utilization efficiency in chickens [6, 25–27], although other studies have also reported that probiotics have no effect on growth performance [28–30]. In contrast, *Clostridium butyricum* is a probiotic that has been shown to improve growth performance and nutrient utilization efficiency in chickens [20, 21, 31] although Zhang et al. reported that *C. butyricum* had no effect on broiler performance [16]. In this study we showed that *C. butyricum* improved the BW and ADG of chickens challenged with *E. coli* K88 compared with broilers in the PC group, and the broilers in the CB group showed no significant differences compared to the CS groups on the BW and ADG overall.

Previous reports had shown that probiotics stimulate the immune response [20, 27, 32–34]. Specifically, *C. butyricum* has been shown as capable of influencing the host immune system by modulating cytokine expression [19, 35–37]. *C. butyricum* could induce the sensitization of the host by increasing pro-inflammatory cytokines such as IL-8, IL-6, and TNF-α, and provide beneficial effects to the host by synthesizing the immunosuppressive cytokines such as IL-10 [19, 36, 37]. IL-4 and TNF-α were also secreted by an intracellular signaling cascade in the immune response in response to *C. butyricum* [38]. Huang et al. [39] reported that *Bacillus* induced TNF-α in spleens and mesenteric lymph nodes of mice [39]. Lee et al. [30] showed that IL-4 transcripts were increased by *B. subtilis* strains LSSAO1, Bs278, and Avicorr in broiler chickens [30]. However, Fujiwara et al. [28] reported that supplementation with *Bacillus subtilis* var. natto fermented soybean did not affect IL-4 gene expression in spleens in broiler chickens [28]. In the present study, chickens fed with *C. butyricum* had higher concentrations of TNF-α and IL-4. This indicated that *C. butyricum* influenced the immune response in broiler chickens challenged with *E. coli* K88.

Lipopolysaccharide (LPS) is an integral component of the outer cell membranes of Gram-negative bacteria which are shed from the bacteria when cell lysis occurs [40]. *E. coli* K88 produces LPS [41], which induced endotoxic shock by triggering the systemic inflammatory response [42–45]. The endotoxins from LPS induce a degenerative morphology and the destruction of lymphocytes in birds [46]. Moreover, endotoxin is associated with intestinal permeability [47–49].When gut permeability is increased, the endotoxin will translocate from the gut into circulation. Ait-Belgnaoui et al. [48] reported that *L. farciminis* treatment prevented stress-induced peripheral endotoxin in rats [48]. DAO is localized mainly in the small intestinal mucosa, particularly near the tips of villi and reflects small intestinal integrity and maturity [50–52]. Intestinal mucosal damage causes leakage of DAO from small intestinal villus tips into the circulation, so DAO is an index of intestinal mucosal barriers [52–54]. Zhang et al [35] reported that the serum level of DAO in allergic mice was markedly higher than that in control mice [35]. Synbiotic therapy prevented HR-related decrease of intestinal integrity that was indicated by the reduction in serum DAO activity [55] and returned serum DAO activities to normal levels after hepatectomy in humans [56]. Zhou et al. reported that *Lactobacillus plantarum* significantly lowered the plasma DAO activities in the bile duct ligation rat model [57]. Sun et al [58] reported that probiotics relatively decreased the levels of LPS and DAO in rats compared with the cardiopulmonary bypass (CPB)-operated ones [58]. In this study, dietary supplementation of *C. butyricum* decreased serum endotoxin and DAO in *E. coli*-challenged birds. Our results indicated that CB benefits the intestinal barrier function in broiler chickens challenged with *E.coli* K88.

The length of villi and the depth of crypt are the important morphological parameters, and are considered as indicators of optimal intestinal functions. The previous studies showed that dietary supplementation with probiotics increased the villus height and villus height: crypt depth ratio, decreased the crypt depth in broiler chickens [59–64]. Our previous research also showed that birds fed *C. butyricum* had higher ileal villus height and lower ileal crypt depth [31]. The present study showed that dietary supplementation with CB increased the jejunal villus height and decreased the crypt depth in broiler chickens challenged with *E.coli* K88. The current result demonstrated that *C. butyricum* improved the structure and function of intestinal mucosa in *E.coli* infected condition.

Amylase, lipase, and protease play very important roles in the digestion of nutrient materials. Reports on the efficacy of probiotics on the digestive enzymes have been varied. Rajput et al. [34, 65] reported that *Saccharomyces boulardii* supplementation increased the activity of lipase, but had no significant improvement in amylase and trypsin in the jejunum of broiler chickens [65]. Wang and Gu [26] reported that the probiotic *Bacillus coagulans* NJ0516 increased the activities of protease and amylase but had no effect on the activities of lipase in broilers [26]. However, de Lima et al. [29] reported that the addition of the probiotic *Bacillus subtilis* in the diet did not affect digestive enzymes activities in broiler chickens [29]. The present study indicated that dietary supplementation of *C. butyricum* promoted digestive enzyme activities in broiler chickens challenged with *E. coli* K88. This was likely due to *C. butyricum*-induced protection of the intestinal integrity by inhibiting the activities of *E. coli* K88 and LPS, so augmented the activities of these enzymes. It is also possible that *C. butyricum* is capable of directly producing digestive enzymes in the gut of animals.

## Conclusion

The results of our study indicated that *E. coli* K88 challenge lowered the BW and ADG, decreased the intestinal barrier function and digestive enzyme activities, but dietary supplementation of *C. butyricum* reversed these observations and promoted the immune response, improved intestinal barrier function and digestive enzyme activities in broiler chickens challenged with *E. coli* K88. Our results suggest that including *C. butyricum* in poultry diets has the potential for rearing healthier birds. There was no significant difference between the *C. butyricum* probiotic treatment and the colistin sulfate antibiotic treatment on the effect of growth performance, immune response, intestinal barrier function, and digestive enzyme activity in broiler chickens challenged with *Escherichia coli*. Therefore, the *C. butyricum* probiotic may be an alternative to antibiotic for animal production.

## Abbreviations

*C. butyricum*: *Clostridium butyricum*
*E. coli* K88: *Escherichia coli* K88
NC: negative control treatment
PC: positive control treatment
CB: *C. butyricum* probiotic treatment
CS: Colistin sulfate antibiotic treatment
BW: body weight
ADG: average day gain
TNF-α: tumor necrosis factor-α
IL-4: interleukin-4
DAO: Diamine oxidase
